# Severe immune dysregulation with neurological impairment and minor bone changes in a child with spondyloenchondrodysplasia due to two novel mutations in the ACP5 gene

**DOI:** 10.1186/1546-0096-13-S1-P141

**Published:** 2015-09-28

**Authors:** H Girschick, C Wolf, H Morbach, C Hertzberg, MA Lee-Kirsch

**Affiliations:** 1 vivantes childrens Hospital Berlin, Pediatrics, Berlin, Germany

## 

**Spondyloenchondrodysplasia (SPENCD)** is a rare skeletal dysplasia, characterized by metaphyseal lesions, neurological impairment and immune dysregulation associated with lupus-like features. SPENCD is caused by biallelic mutations in the *ACP5* gene encoding tartrate-resistant phosphatase. We report on a child, who presented with spasticity, multisystem inflammation, autoimmunity and immunodeficiency with minimal metaphyseal changes due to compound heterozygosity for two novel *ACP5* mutations. These findings extend the phenotypic spectrum of SPENCD and indicate that *ACP5* mutations can cause severe immune dysregulation and neurological impairment even in the absence of metaphyseal dysplasia.

